# Reply to Freckmann et al. Comment on “Hochfellner et al. Accuracy Assessment of the GlucoMen^®^ Day CGM System in Individuals with Type 1 Diabetes: A Pilot Study. *Biosensors* 2022, *12*, 106”

**DOI:** 10.3390/bios13070710

**Published:** 2023-07-05

**Authors:** Daniel A. Hochfellner, Amra Simic, Marlene T. Taucher, Lea S. Sailer, Julia Kopanz, Tina Pöttler, Julia K. Mader

**Affiliations:** Division of Endocrinology and Diabetology, Department of Internal Medicine, Medical University of Graz, 8036 Graz, Austria; daniel.hochfellner@medunigraz.at (D.A.H.); amra.ajsic@medunigraz.at (A.S.); marlenetaucher@gmail.com (M.T.T.); lea.sailer02@gmail.com (L.S.S.); juliakopanz@gmx.at (J.K.); tina.poettler@medunigraz.at (T.P.)

We thank Dr. Freckmann et al. [1] for the comment and for the detailed review of our data.

Referring to the first point about the correction of lag time, we agree that the chosen approach has some limitations in the interpretation of the results. This aspect can be discussed together with the second point concerning mean absolute relative difference (MARD) data representation. The main aim of this study was to conduct an evaluation of the GlucoMen^®^ Day CGM system, which had recently been launched on the market by A. Menarini Diagnostics based on available data related to the CE registration study. For this reason, we limited the number of enrolled participants to just eight, resulting in limited statistics, and the methods and metrics for analysis were chosen according to the same methods used in the system CE registration study [2].

Thus, actually, the results met the predefined accuracy criteria of the trial, but we can agree that they cannot be consistently compared with other research on CGM accuracy assessment. In order to avoid any confusion, such results can be better clarified as follows: when comparing CGM to PG, the MARD for values ≥ 100 mg/dL was 9.7 [2.6–14.6]%. The MARD for values ≥ 100 mg/dL for CGM vs. SMBG was 13.1 [3.5–18.6]%. The mean absolute difference (MAD) for glucose values < 100 mg/dL was 20.5 ± 18.7 mg/dL (vs. PG) and 16.6 ± 16.8 mg/dL (vs. SMBG).

The preference of MAD for lower glycemic intervals has mainly a mathematical reason: the relative (percentage) bias between a measurement and its reference increases the lower the reference value. Based on this principle, for example, ISO 15197:2015 for BGMs specifies to use an absolute bias for values below 100 mg/dL and % bias at values higher or equal to 100 mg/dL. Such considerations are also widely discussed in the literature [3]. Therefore, also given the small sample size of this study, MAD was considered to be more appropriate for lower glucose concentration ranges, while the chosen cut-off of 100 mg/dL was derived for comparison purposes with available data for this system and other CGM studies [2,4].

To conclude, the present analysis confirmed that the GlucoMen^®^ Day CGM is a user- and environmentally friendly system with performance in line with previously reported accuracy criteria. In future studies, the system will be more extensively evaluated based on current metrics to determine whether it meets the clinical requirements for state-of-the-art CGMs and user expectations.
